# Alcohol use and poor sleep quality: a longitudinal twin study across 36 years

**DOI:** 10.1093/sleepadvances/zpac023

**Published:** 2022-07-06

**Authors:** Viola Helaakoski, Jaakko Kaprio, Christer Hublin, Hanna M Ollila, Antti Latvala

**Affiliations:** Institute for Molecular Medicine Finland, HiLIFE, University of Helsinki, Helsinki, Finland; Institute for Molecular Medicine Finland, HiLIFE, University of Helsinki, Helsinki, Finland; Finnish Institute of Occupational Health, Helsinki, Finland; Department of Public Health, Faculty of Medicine, University of Helsinki, Helsinki, Finland; Institute for Molecular Medicine Finland, HiLIFE, University of Helsinki, Helsinki, Finland; Broad Institute of MIT and Harvard, Cambridge, Massachusetts, USA; Center for Genomic Medicine, Massachusetts General Hospital, Boston, USA; Anesthesia, Critical Care and Pain Medicine, Massachusetts General Hospital and Harvard Medical School, Boston, USA; Institute of Criminology and Legal Policy, University of Helsinki, Helsinki, Finland

**Keywords:** alcohol consumption, sleep quality, longitudinal study, twin study

## Abstract

**Study Objectives:**

Poor sleep is one of the multiple health issues associated with heavy alcohol consumption. While acute effects of alcohol intake on sleep have been widely investigated, the longitudinal associations remain relatively underexplored. The objective of our research was to shed light on cross-sectional and longitudinal associations between alcohol use and poor sleep quality over time, and to elucidate the role of familial confounding factors in such associations.

**Methods:**

Using self-report questionnaire data from the Older Finnish Twin Cohort (*N* = 13 851), we examined how alcohol consumption and binge drinking are associated with sleep quality during a period of 36 years.

**Results:**

Cross-sectional logistic regression analyses revealed significant associations between poor sleep and alcohol misuse, including heavy and binge drinking, at all four time points (OR range = 1.61–3.37, *p* < .05), suggesting that higher alcohol intake is associated with poor sleep quality over the years. Longitudinal cross-lagged analyses indicated that moderate, heavy and binge drinking predict poor sleep quality (OR range = 1.25–1.76, *p* < .05), but not the reverse. Within-pair analyses suggested that the associations between heavy drinking and poor sleep quality were not fully explained by genetic and environmental influences shared by the co-twins.

**Conclusions:**

In conclusion, our findings support previous literature in that alcohol use is associated with poor sleep quality, such that alcohol use predicts poor sleep quality later in life, but not vice versa, and that the association is not fully explained by familial factors.

**Statement of Significance**

Poor sleep is a symptom of insomnia, which affects approximately 20% of the general population and is associated with an increased risk of disease. The association between poor sleep and alcohol use has been well established, but the longitudinal relationship remains unclear. We analyzed the associations between poor sleep and drinking over 36 years in a twin cohort of 13 851 participants. We found that increased alcohol use is cross-sectionally associated with poor sleep, and more importantly, predicts poor sleep later in life, but not vice versa. Furthermore, our findings suggest that familial factors do not fully account for these associations. These results highlight the potential adverse longitudinal effects of alcohol on sleep, and thereby, on overall health.

## Introduction

Sleep is a vital, often undervalued, element of mental and physical health. Complaints of poor sleep among other sleeping problems have become increasingly common among the general population. Poor sleep is a symptom of insomnia which affect approximately 20% of the general population on an acute, recurring or chronic basis [1–3]. Insomnia is one of the most common reasons for sleeping problems, and is characterized by difficulties with falling asleep, staying asleep or waking up too early, in addition to complaints of nonrestorative sleep. Insomnia generally results in some form of daytime impairment through e.g. fatigue and mood changes. Chronic insomnia is associated with an increased risk of various cardiovascular, autoimmune and psychiatric diseases [4–6]. The causes of insomnia are multifactorial, ranging from genetic influences to different lifestyle factors. A major lifestyle factor associated with insomnia symptoms is alcohol misuse, including heavy alcohol consumption and binge drinking [7]. Because many of the brain systems and neurotransmitters associated with sleep–wake regulation are also influenced by alcohol intake, it is biologically credible that drinking affects sleep [8].

Some individuals ‘self-medicate’ their sleep problems with alcohol [9], even though the negative effects of a single pre-bedtime dose of alcohol on sleep are well known. Previous research suggests that alcohol, through its sedative effect, may initially shorten sleep onset latency at all dosages, but disrupt the quality of sleep in the second half of the night. High doses of alcohol appear to significantly suppress rapid eye movement (REM) sleep in the first half of the night, and the total amount of REM sleep tends to be decreased at moderate and high doses. Multiple studies suggest that the onset of the first REM sleep stage is considerably delayed at all doses. Alcohol at all dosages also tends to reduce wakefulness in the first half of sleep, but is likely to increase it in the second part of the night in accordance with the metabolic elimination of ethanol from the body [7, 10]. Furthermore, previous literature suggests that slow wave sleep (SWS) tends to increase in the first half of the night at all doses, whereas total night SWS increases at high doses of alcohol [10].

Previous literature has mainly focused on short-term effects of alcohol on sleep quality, whereas studies on the long-term effects remain somewhat limited. Some studies suggests that sleep problems might predict increased drinking and the development of alcohol-related problems [11], whereas other studies have found that persistent heavy drinking over several decades is associated with increased sleep disturbances [12]. Thus, more research is needed to clarify whether heavy drinking predicts sleep disruption, or whether it is sleep disruption that increases the risk of alcohol misuse, or whether both effects exist.

Regarding alcohol use disorder (AUD), research consistently indicates a high comorbidity with insomnia, with a large proportion of AUD patients reporting insomnia symptoms either whilst drinking or during recovery [13, 14]. It is common for individuals with AUD to develop poor sleep hygiene with an irregular sleep schedule, napping during the day and greater wakefulness at night. On the other hand, poor sleeping habits and insomnia symptoms may in some cases precede AUD, thereby suggesting a potential bidirectional relationship between insomnia and AUD [15]. It should be noted, though, that the development of AUD is typically a result of long-term heavy drinking, and so sleep problems might develop during the subclinical phase of AUD. The chronic effects and directionality between sleep disruption and alcohol use remains less clear in healthy individuals.

The aim of the current research was to expand our understanding of the relationship between alcohol misuse and poor sleep, and to examine how genetic and familial factors might contribute to this relationship. We investigated how heavy alcohol consumption and binge drinking during adulthood are associated with poor sleep quality across a period of 36 years. Understanding these associations is crucial for developing effective preventive and treatment strategies for both sleep and alcohol disorders.

## Methods

### Data

We used data from the Older Finnish Twin Cohort [16], which includes Finnish monozygotic (MZ) and same-sex dizygotic (DZ) twin pairs born before the year of 1958. The baseline survey took place in 1975, with three follow-up health and lifestyle surveys in 1981, 1990, and 2011. The 1990 survey was completed by twins born in 1930–1957, and the 2011 by twins born in 1945–1957. Only participants born in 1945–1957 were included in our analyses (*N* = 13 851), so as to control for age differences and exclude those who did not have a chance to take part in all four surveys. The effective sample size varied somewhat during the follow-up and in different analyses, and exact numbers of participants are given along with the results below. Ethical permissions for the Older Twin Cohort surveys were obtained from the Ethics Committee of the Department of Public Health, University of Helsinki and the Ethical Committee of the University Hospital District of Helsinki and Uusimaa.

### Measures

As for sleep variables, we focused on poor sleep quality. In addition, we examined associations with short sleep duration as a sensitivity analysis, as short sleep correlates both with sleep quality and insomnia symptoms. Trends in sleep quality and duration over time in the same cohort have already been extensively investigated [17, 18]. The items included in all four surveys, with the distributions for each trait at each time point are reported in Table 1. Sleep quality was measured by asking participants whether they tend to sleep: “well”, “fairly well”, “fairly poorly” or “poorly”. For the purposes of our analyses, cases of poor sleep were coded to include those who reported sleeping either “fairly poorly” or “poorly”, whilst those reporting sleeping “well” or “fairly well” were coded as sleeping well. Sleep duration was coded as short sleep (<7 h), average sleep (7–8 h) and long sleep (>8 h). Analyses of short sleep duration were restricted to those reporting short sleep with average sleepers coded as a baseline category, whilst long sleepers were excluded from the analyses.

**Table 1.** Participant characteristics and distributions of relevant sleep and drinking categories.

Total *N* = 13 851*	All				Men				Women			
	1975 (*N* = 12 660)	1981 (*N* = 12 297)	1990 (*N* = 7724)	2011 (*N* = 8408)	1975 (*N* = 6220) (49.1%)	1981 (*N* = 5910) (48.1%)	1990 (*N* = 3434) (44.4%)	2011 (*N* = 3753) (44.6%)	1975 (*N* = 6440) (50.9%)	1981 (*N* = 6387) (51.9%)	1990 (*N* = 4290) (55.6%)	2011 (*N* = 4655) (55.4%)
Mean age in years (SD)	23.9 (3.8)	30.1 (3.8)	39.2 (3.8)	60.3 (3.8)	24.0 (3.6)	30.3 (3.7)	39.4 (3.7)	60.4 (3.7)	23.7 (3.7)	30.0 (3.8)	39.0 (3.8)	60.1 (3.8)
Mean BMI (SD)	21.8 (2.7)	22.7 (3.0)	24.0 (3.7)	26.2 (4.3)	22.6 (2.6)	23.7 (2.8)	24.9 (3.2)	26.7 (3.9)	20.9 (2.6)	21.7 (2.9)	23.3 (3.9)	25.8 (4.6)
Current smoker	4929 (38.9%)	4312 (35.1%)	2226 (28.8%)	1499 (17.8%)	2789 (44.8%)	2516 (42.6%)	1166 (31.1%)	740 (19.7%)	2140 (33.2%)	1796 (28.1%)	1060 (24.7%)	759 (16.3%)
Mean life satisfaction score (SD)	8.6 (2.9)	8.5 (2.9)	8.5 (3.0)	8.0 (3.0)	8.7 (2.9)	8.6 (2.9)	8.5 (2.9)	8.0 (2.9)	8.5 (3.0)	8.4 (2.9)	8.5 (3.0)	8.1 (3.1)
Sleep quality categories												
Sleeping well	6471 (51.1%)	5928 (48.2%)	4275 (55.3%)	1887 (22.4%)	3288 (52.9%)	2855 (48.3%)	1849 (53.8%)	980 (26.1%)	3183 (49.4%)	3073 (48.1%)	2426 (56.6%)	907 (19.5%)
Sleeping fairly well	5463 (43.2%)	5501 (44.7%)	2185 (28.3%)	4815 (57.3%)	2556 (41.1%)	2602 (44.0%)	995 (29.0%)	2119 (56.5%)	2907 (45.1%)	2899 (45.4%)	1190 (27.7%)	2696 (57.9%)
Sleeping fairly poorly	454 (3.6%)	610 (5.0%)	828 (10.7%)	1256 (14.9%)	228 (3.7%)	319 (5.4%)	390 (11.4%)	489 (13.0%)	226 (3.5%)	291 (4.6%)	438 (10.2%)	767 (16.5%)
Sleeping poorly	97 (0.8%)	136 (1.1%)	412 (5.3%)	346 (4.1%)	50 (0.8%)	73 (1.2%)	187 (5.4%)	120 (3.2%)	47 (0.7%)	63 (1.0%)	225 (5.2%)	226 (4.9%)
**Sleep duration categories**												
Short (<7 h)	1020 (8.1%)	1600 (13.0%)	1364 (17.7%)	1936 (23.0%)	584 (9.4%)	885 (15.0%)	704 (20.5%)	868 (23.1%)	436 (6.8%)	715 (11.2%)	660 (15.4%)	1068 (22.9%)
Average (7-8 h)	9565 (75.6%)	8401 (68.3%)	5226 (67.7%)	5270 (62.7%)	4779 (76.8%)	4167 (70.5%)	2347 (68.3%)	2351 (62.6%)	4786 (74.3%)	4234 (66.3%)	2879 (67.1%)	2919 (62.7%)
Long (>8 h)	2011 (15.9%)	2244 (18.2%)	1107 (14.3%)	1166 (13.9%)	821 (13.2%)	834 (14.1%)	370 (10.8%)	518 (13.8%)	1,190 (18.5%)	1410 (22.1%)	737 (17.2%)	648 (13.9%)
**Alcohol drinking categories**												
Abstainer	1663 (13.1%)	1508 (12.3%)	977 (12.6%)	574 (6.8%)	633 (10.2%)	456 (7.7%)	276 (8.0%)	178 (4.7%)	1030 (16.0%)	1052 (16.5%)	701 (16.3%)	396 (8.5%)
Light drinker	4881 (38.6%)	5002 (40.7%)	2679 (34.7%)	3215 (38.2%)	1550 (24.9%)	1557 (26.3%)	730 (21.3%)	906 (24.1%)	3331 (51.7%)	3445 (53.9%)	1949 (45.4%)	2309 (49.6%)
Moderate drinker	4545 (35.9%)	4240 (34.5%)	2900 (37.5%)	2604 (31.0%)	2982 (47.9%)	2867 (48.5%)	1755 (51.1%)	1685 (44.9%)	1563 (24.3%)	1373 (21.5%)	1145 (26.7%)	919 (19.7%)
Heavy drinker	1512 (11.9%)	1508 (12.3%)	1147 (14.8%)	1485 (17.7%)	1023 (16.4%)	1018 (17.2%)	662 (19.3%)	812 (21.6%)	489 (7.6%)	490 (7.7%)	485 (11.3%)	673 (14.5%)
Non-binge drinker	8734 (69.0%)	8363 (68.0%)	5365 (69.5%)	5823 (69.3%)	3248 (52.2%)	2920 (49.4%)	1748 (50.9%)	2173 (57.9%)	5486 (85.2%)	5443 (85.2%)	3617 (84.3%)	3650 (78.4%)
Binge drinker	3858 (30.5%)	3833 (31.2%)	2284 (29.6%)	2029 (24.1%)	2934 (47.2%)	2945 (49.8%)	1648 (48.0%)	1396 (37.2%)	924 (14.3%)	888 (13.9%)	636 (14.8%)	633 (13.6%)

*Total *N* includes all participants who have taken at least one of the four surveys and answered some, but not necessarily all, of the questions of that survey. Percentages of distributions are calculated based on the N of each questionnaire.

Regarding alcohol measures, we investigated the amount of alcohol intake as well as binge drinking. Trends in drinking categories in the Older Finnish Twin Cohort have also been investigated by previous research [19]. Alcohol intake was measured by the monthly amount of alcohol consumed (in grams). Participants were categorized as “heavy drinkers”, “moderate drinkers”, “light drinkers” and “abstainers”. Drinking levels were defined as per the NIAAA guidelines [20], so that weekly consumption was calculated based on monthly consumption, as in previous studies of the same cohort [19, 21]. Heavy drinkers include those who reported drinking more than seven drinks per week (336 g/month) for women and more than 14 drinks per week (672 g/month) for men. Moderate drinkers include participants drinking more than 3 drinks but less than 7 drinks per week (145–336 g/month) for women and less than 14 drinks per week (145–672 g/month) for men. Light drinkers refer to those drinking less than 3 drinks per week (1–144 g/month). Finally, abstainers refer to participants who reported consuming no alcohol at all at the time of taking each survey. As we were interested in increased alcohol use, we used light drinking as a baseline category. As for binge drinking, the participants were asked whether they drank more than 5 bottles of beer, 1 bottle of wine or 4 drinks (≥18 ml of spirits) on the same occasion at least once a month. Those who were defined as abstainers at the time of taking each survey were omitted from the analyses, because the association between sleep and abstinence is likely to reflect different factors compared to the amount of alcohol consumed (e.g. chronic illness, long-term medication or past AUD).

### Covariates

We included body mass index (BMI), smoking and life satisfaction as covariates in the analyses. BMI was based on self-reported measures of weight in kilograms and height in centimeters at each survey. Smoking refers to current smoking status (yes/no) at the time of taking each survey.

Life satisfaction (LS) refers to a 4-item summary variable which comprises questions regarding how interesting, happy, easy and lonely the participant rates their life at present. LS has previously been correlated with e.g. depressive symptoms in the same cohort [22]. Associations between LS and affective, anxiety, and other psychiatric disorders have also been established in other cohorts [23, 24], and so LS can be used as a proxy to estimate overall mental health in self-reported questionnaires where clinical diagnoses are not available. Previous research also suggests that life satisfaction and heavy alcohol consumption predict each other [25], and so adjusting for LS can be used to adjust for potential confounding. The total score ranged from 4 (high LS) to 20 (low LS). The sum score was calculated allowing one missing item. LS was available at each survey.

### Statistical analysis

After running descriptive statistics, we conducted logistic regression analyses to investigate cross-sectional associations between the sleep and alcohol traits. Using cluster correction, we adjusted the analyses for sampling of twin individuals as twin pairs [26]. As for covariates, all analyses were first adjusted for age and sex (Model 1). We then performed the same analyses by including BMI, smoking and LS as additional covariates (Model 2) to rule out possible confounding by these factors. We also conducted interaction analyses to examine sex differences. After this we used a cross-lagged path model to investigate the predictive associations between sleep and alcohol traits over time. In order to have both the alcohol and the sleep variables as binary in the cross-lagged models, we combined the moderate and heavy drinking categories into one and used light drinking as the reference category, similar to the cross-sectional analysis.

Finally, we conducted fixed effects within-pair analyses to examine associations within twin pairs, MZ and DZ both separately and together, so as to account for familial (i.e., genetic and shared environmental) confounding [27]. By design, these analyses rule out factors that are constant within twin pairs, i.e. all genetic and environmental factors that are shared between co-twins. As MZ twins share 100% of their genome, whereas DZ twins share, on average, 50% of their segregating genes, and both twin types share their rearing environment equally, the comparison of results within MZ vs. DZ twin pairs is informative on the contribution of genetic and shared environmental influences. Specifically, an association similarly attenuated within MZ and DZ pairs, as compared to the individual-level association, suggests a contribution of shared environmental factors whereas gradual attenuation of associations between individual level, DZ and MZ pairs, accounting for 0%, 50% and 100% of genetic influences, respectively, suggests genetic effects [28].

As an additional analysis, to illuminate the effects of age vs. cohort/period on the cross-sectional associations between alcohol use and sleep measures, we re-ran the analyses for data from the 1975 and 1981 study waves among participants born in 1930–1942 and participants born in 1915–1927, respectively, so that the age ranges matched with the original cohort (i.e. twins born 1945–1957) in the 1990 and 2011 questionnaires.

All analyses were conducted using Stata17 (StataCorp. 2021. *Stata Statistical Software: Release 17*. College Station, TX: StataCorp LLC).

## Results

### Descriptive statistics

Table 1 shows the distributions of the sleep and alcohol variables as well as covariates across study waves. The average age of participants at baseline was 23.7 years in women and 24 years in men. Gender distributions at baseline were 50.9% female and 49.1% male. Sleep quality appeared to decline in both genders with age over the years, whereas men appeared to sleep slightly better compared to women. On the other hand, the majority of participants slept well or fairly well throughout the observation period, as has been reported earlier. Sleep duration decreased in both men and women during the 36-year follow-up, whereas most participants reported sleeping 7–8 h per night at all time points. As for alcohol consumption, drinking quantities increased and the proportion of abstainers decreased over the years. Heavy drinking increased in both genders, whereas trends in binge drinking were somewhat inconsistent. Overall, women appeared to drink substantially less than men.

### Cross-sectional associations between sleep and drinking traits

Including sex and age as covariates (Model 1), cross-sectional analyses between drinking and sleep traits revealed associations between heavy and binge drinking and poor sleep at all time points, with Odds Ratios (ORs) ranging from 1.61 to 3.37 (Table 2). The strongest association was observed between heavy drinking and poor sleep quality in 1981 (OR = 3.37 [95% CI: 2.74, 4.14]) when the average age of twins was 30 years. Heavy and binge drinking were also associated with short sleep at all time points. At earlier time points, moderate drinking, as compared to light drinking, was also associated with both poor and short sleep, with ORs ranging from 1.18 to 1.30.

**Table 2.** Cross-sectional associations between drinking categories (predictor variables) and sleep traits (outcome variables).

	Model 1			Model 2		
	Odds ratio	[95% CI]	*P*-value	Odds ratio	[95% CI]	*P*-value
**Poor sleep**						
**1975 (*N* = 12 485)**						
**Abstainer**	0.94	[0.68, 1.29]	.70	1.20	[0.66, 2.16]	.55
**Light**	1.00 (baseline)	–	–	1.00 (baseline)	–	–
**Moderate**	1.29	[1.03, 1.60]	.025	1.27	[0.93, 1.74]	.13
**Heavy**	2.93	[2.31, 3.72]	<.001	2.33	[1.69, 3.22]	<.001
**Non-binge**	1.00 (baseline)			1.00 (baseline)		
**Binge**	2.13	[1.77, 2.58]	<.001	1.67	[1.31, 2.13]	<.001
**1981 (*N* = 12 175)**						
**Abstainer**	1.06	[0.80, 1.40]	.68	1.39	[0.92, 2.10]	.12
**Light**	1.00 (baseline)	–	–	1.00 (baseline)	–	–
**Moderate**	1.30	[1.07, 1.58]	.0082	1.04	[0.81, 1.33]	.78
**Heavy**	3.37	[2.74, 4.14]	<.001	2.12	[1.63, 2.77]	<.001
**Non-binge**	1.00 (baseline)			1.00 (baseline)		
**Binge**	2.19	[1.85, 2.59]	<.001	1.56	[1.26, 1.92]	<.001
**1990 (*N* = 7700)**						
**Abstainer**	1.24	[1.00, 1.55]	.051	1.59	[1.09, 2.32]	.017
**Light**	1.00 (baseline)	–	–	1.00 (baseline)	–	–
**Moderate**	1.25	[1.06, 1.47]	.0084	1.29	[1.01, 1.64]	.039
**Heavy**	3.02	[2.51, 3.62]	<.001	2.66	[2.06, 3.43]	<.001
**Non-binge**	1.00 (baseline)			1.00 (baseline)		
**Binge**	2.26	[1.96, 2.60]	<.001	1.93	[1.60, 2.34]	<.001
**2011 (*N* = 8304)**						
**Abstainer**	1.28	[1.03, 1.60]	.029	1.11	[0.77, 1.58]	.58
**Light**	1.00 (baseline)	–	–	1.00 (baseline)	–	–
**Moderate**	1.06	[0.92, 1.22]	.45	1.16	[0.94, 1.44]	.17
**Heavy**	1.78	[1.53, 2.07]	<.001	1.76	[1.42, 2.19]	<.001
**Non-binge**	1.00 (baseline)			1.00 (baseline)		
**Binge**	1.61	[1.37, 1.88]	<.001	1.55	[1.31, 1.85]	<.001
**Short sleep**						
**1975 (*N* = 10 585)**						
**Abstainer**	0.86	[0.67, 1.12]	.26	1.55	[1.04, 2.31]	.030
**Light**	1.00 (baseline)	–	–	1.00 (baseline)	–	–
**Moderate**	1.26	[1.07, 1.49]	.0057	1.00	[0.82, 1.23]	.97
**Heavy**	2.14	[1.75, 2.60]	<.001	1.27	[0.99, 1.61]	.055
**Non-binge**	1.00 (baseline)			1.00 (baseline)		
**Binge**	1.71	[1.47, 1.97]	<.001	1.14	[0.95, 1.36]	.17
**1981 (*N* = 10 001)**						
**Abstainer**	0.93	[0.76, 1.15]	.51	1.038	[0.74, 1.45]	.83
**Light**	1.00 (baseline)	–	–	1.00 (baseline)	–	–
**Moderate**	1.18	[1.03, 1.35]	.016	0.97	[0.81, 1.14]	.69
**Heavy**	1.84	[1.56, 2.18]	<.001	1.31	[1.07, 1.61]	.0080
**Non-binge**	1.00 (baseline)			1.00 (baseline)		
**Binge**	1.45	[1.28, 1.64]	<.001	1.14	[0.98, 1.32]	.086
**1990 (*N* = 6590)**						
**Abstainer**	0.91	[0.73, 1.13]	.38	0.91	[0.62, 1.34]	.64
**Light**	1.00 (baseline)	–	–	1.00 (baseline)	–	–
**Moderate**	0.97	[0.83, 1.13]	.66	0.84	[0.68, 1.04]	.11
**Heavy**	1.41	[1.17, 1.69]	<.001	0.98	[0.77, 1.25]	.87
**Non-binge**	1.00 (baseline)			1.00 (baseline)		
**Binge**	1.46	[1.27, 1.68]	<.001	1.09	[0.90, 1.32]	.36
**2011 (*N* = 7206)**						
**Abstainer**	1.29	[1.04, 1.60]	.021	1.19	[0.85, 1.66]	.30
**Light**	1.00 (baseline)	–	–	1.00 (baseline)	–	–
**Moderate**	1.00	[0.87, 1.14]	.96	1.03	[0.85, 1.26]	.75
**Heavy**	1.26	[1.08, 1.47]	.0033	1.20	[0.97, 1.48]	.086
**Non-binge**	1.00 (baseline)			1.00 (baseline)		
**Binge**	1.34	[1.19, 1.50]	<.001	1.19	[1.01, 1.40]	.038

Model 1 includes sex and age as covariates, whereas Model 2 was adjusted for sex, age, BMI, smoking and life satisfaction. Statistically significant (*p* < .05) associations are highlighted in red.

Further adjusting for BMI, smoking and LS (Model 2), associations between heavy/binge drinking and poor sleep quality mostly remained of similar effect sizes as in Model 1, whereas associations with short sleep duration were attenuated (Table 2). Associations between moderate drinking and sleep traits were similarly attenuated. Interaction analyses revealed no statistically significant sex differences in the associations.

In the additional analyses on the effects of age vs. cohort/period on the cross-sectional associations, results from participants born in 1930–1942 in 1975 were statistically less significant with effect sizes being slightly smaller for poor sleep and slightly bigger for short sleep, as compared to results from the main study cohort in 1990. Results from participants born in 1915–1927 in 1981 were nonsignificant apart from associations with binge drinking where Odds Ratios were slightly smaller for poor sleep and slightly bigger for short sleep, as compared with the original cohort in 2011 (Supplementary Table S1).

### Cross-lagged associations between sleep and drinking traits

**Figure 1:**
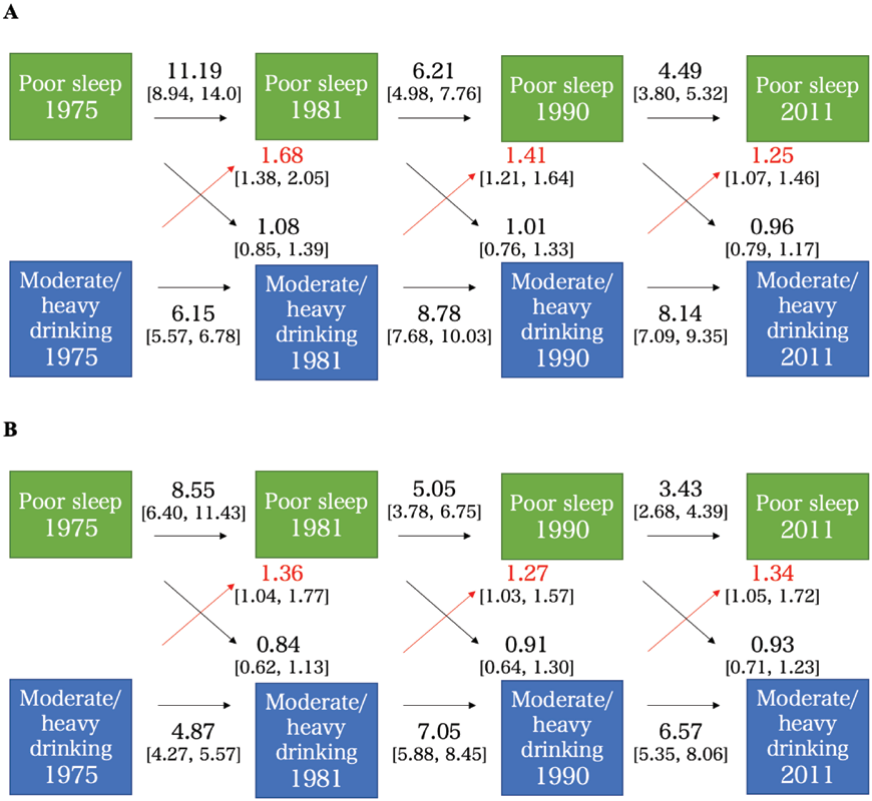
(A) Cross-lagged associations between moderate/heavy drinking and poor sleep quality, adjusting for sex and age (Model 1). The figure shows odds ratios and 95% confidence intervals for each association, with statistically significant (*p* < .05) associations between drinking and sleep traits highlighted in red. (B) Cross-lagged associations between moderate/heavy drinking and poor sleep quality, adjusting for sex, age, BMI, smoking and life satisfaction (Model 2). The figure shows odds ratios and 95% confidence intervals for each association, with statistically significant (*p* < .05) associations between drinking and sleep traits highlighted in red.

Poor sleep at earlier time points predicted poor sleep at later time points across all study waves, and a similar pattern was seen with heavy drinking. Poor sleep was not associated with heavy drinking at subsequent time points, but heavy drinking predicted subsequent poor sleep (Figure 1A). We observed similar, and even slightly stronger associations with binge drinking, so that earlier binge drinking predicted later poor sleep (Supplementary Figure S1A). We then adjusted the models with BMI, smoking and LS assessed at each time point. A very similar pattern from heavy drinking to poor sleep can be seen in both models (Figure 1B). As for binge drinking, the estimates remained consistent after adjusting for other covariates, although not all associations remained statistically significant, suggesting a role of other environmental and lifestyle factors (Supplementary Figure S1B). Results for short sleep were somewhat inconsistent and statistically nonsignificant after adjusting in Model 2 so that no robust conclusions could be drawn.

### Within-pair models

In within-pair analyses including both MZ and DZ pairs, as shown in Table 3, heavy and binge drinking were associated with an elevated risk of poor sleep quality as compared with light drinking and nonbinge drinking (ORs ranging between 1.69 and 2.58), suggesting the associations were not fully explained by familial factors. Overall, associations with short sleep duration were weaker and mostly nonsignificant. Associations within MZ and DZ twin pairs are given in Supplementary Tables S2A and S2B, respectively. Overall, there were more statistically significant associations within DZ than within MZ pairs, but effect sizes were often of similar magnitude.

**Table 3.** Within-pair associations including both mono- and dizygotic twins.

	Model 1			Model 2		
	Odds ratio	[95% CI]	*P*-value	Odds ratio	[95% CI]	*P*-value
**Poor sleep**						
**1975 (*N* = 764)**						
**Abstainer**	1.12	[0.66, 1.90]	.67	1.74	[0.52, 5.83]	.37
**Light**	1.00 (baseline)			1.00 (baseline)		
**Moderate**	0.97	[0.65, 1.44]	.86	1.23	[0.65, 2.32]	.52
**Heavy**	1.99	[1.17, 3.38]	.011	2.12	[1.00, 4.51]	.050
**Non-binge**	1.00 (baseline)			1.00 (baseline)		
**Binge**	1.70	[1.16, 2.47]	.0059	1.67	[0.98, 2.85]	.061
**1981 (*N* = 916)**						
**Abstainer**	1.39	[0.81, 2.38]	.23	2.62	[0.81, 8.47]	.11
**Light**	1.00 (baseline)			1.00 (baseline)		
**Moderate**	1.25	[0.87, 1.78]	.23	1.72	[0.97, 3.04]	.063
**Heavy**	2.04	[1.31, 3.18]	.0016	2.58	[1.24, 5.36]	.012
**Non-binge**	1.00 (baseline)			1.00 (baseline)		
**Binge**	1.75	[1.24, 2.47]	.0014	1.71	[1.01, 2.91]	.046
**1990 (*N* = 1236)**						
**Abstainer**	1.28	[0.83, 1.97]	.27	0.98	[0.37, 2.60]	.97
**Light**	1.00 (baseline)			1.00 (baseline)		
**Moderate**	1.13	[0.84, 1.52]	0.43	1.22	[0.73, 2.04]	0.45
**Heavy**	2.65	[1.78, 3.95]	<0.001	2.45	[1.33, 4.51]	0.0040
**Non-binge**	1.00 (baseline)			1.00 (baseline)		
**Binge**	2.01	[1.48, 2.72]	<.001	2.05	[1.27, 3.30]	.0033
**2011 (*N* = 1372)**						
**Abstainer**	1.35	[0.87, 2.10]	.19	1.43	[0.59, 3.48]	.43
**Light**	1.00 (baseline)			1.00 (baseline)		
**Moderate**	1.39	[1.03, 1.87]	.032	1.31	[0.75, 2.31]	.35
**Heavy**	1.74	[1.23, 2.45]	.0016	1.80	[1.01, 3.20]	.048
**Non-binge**	1.00 (baseline)			1.00 (baseline)		
**Binge**	1.69	[1.25, 2.30]	<.001	1.38	[0.86, 2.24]	.19
**Short sleep**						
**1975 (*N* = 1186)**						
**Abstainer**	1.06	[0.62, 1.81]	.84	0.89	[0.38, 2.11]	.80
**Light**	1.00 (baseline)			1.00 (baseline)		
**Moderate**	1.28	[0.93, 1.76]	.13	1.47	[0.92, 2.36]	.11
**Heavy**	2.56	[1.64, 4.00]	<.001	3.16	[1.65, 6.05]	<.001
**Non-binge**	1.00 (baseline)			1.00 (baseline)		
**Binge**	1.38	[1.04, 1.84]	.026	1.00	[0.69, 1.46]	.98
**1981 (*N* = 1530)**						
**Abstainer**	0.99	[0.66, 1.50]	.98	1.24	[0.61, 2.52]	.55
**Light**	1.00 (baseline)			1.00 (baseline)		
**Moderate**	1.16	[0.89, 1.51]	.28	0.94	[0.66, 1.35]	.76
**Heavy**	1.76	[1.18, 2.65]	.0061	1.32	[0.78, 2.26]	.30
**Non-binge**	1.00 (baseline)			1.00 (baseline)		
**Binge**	1.25	[0.95, 1.63]	.11	1.15	[0.80, 1.66]	.46
**1990 (*N* = 1110)**						
**Abstainer**	0.74	[0.47, 1.18]	.21	0.36	[0.091, 1.39]	.14
**Light**	1.00 (baseline)			1.00 (baseline)		
**Moderate**	1.04	[0.78, 1.40]	.78	0.89	[0.55, 1.43]	.62
**Heavy**	1.42	[0.92, 2.19]	.11	1.11	[0.59, 2.09]	.74
**Non-binge**	1.00 (baseline)			1.00 (baseline)		
**Binge**	1.37	[0.99, 1.90]	.055	1.54	[0.98, 2.41]	.059
**2011 (*N* = 1328)**						
**Abstainer**	1.38	[0.86, 2.23]	.19	0.94	[0.35, 2.51]	.90
**Light**	1.00 (baseline)			1.00 (baseline)		
**Moderate**	1.17	[0.89, 1.54]	.25	1.12	[0.69, 1.82]	.64
**Heavy**	1.36	[0.95, 1.93]	.091	1.14	[0.64, 2.04]	.66
**Non-binge**	1.00 (baseline)			1.00 (baseline)		
**Binge**	1.15	[0.90, 1.47]	.27	1.14	[0.71, 1.84]	.58

Model 1 includes sex and age as covariates, whereas Model 2 was adjusted for sex, age, BMI, smoking and life satisfaction. Statistically significant (*p* < .05) associations are highlighted in red.

## Discussion

The findings of the current study confirm that heavy and binge drinking are associated with poor sleep quality. Leveraging repeated measurements across 36 years, cross-sectional analyses reveal that heavy and binge drinking are associated with poor sleep at all time points through adulthood. Our findings not only establish a robust association between high volumes of drinking and poor sleep but also suggest that moderate and heavy alcohol consumption predict poor sleep in a longitudinal setting, whereas we found little evidence for the reverse associations.

Our results support the numerous earlier findings suggesting that drinking and sleep are connected (reviewed in e.g. [29, 30]). The immediate effects of alcohol on sleep are well established, suggesting that consuming even relatively small amounts of alcohol before bedtime increases sleep fragmentation and impairs REM sleep [31, 32]. The long-term effects of alcohol on sleep and vice versa require further research. Earlier studies suggest that the relationship of alcohol misuse with sleep is bidirectional. A growing body of literature comprises studies suggesting that insomnia symptoms are associated with subsequent drinking [33, 34], whereas other studies have found alcohol use and dependence to precede insomnia [14, 35]. Results of the current study are consistent with possible effects of binge and heavy drinking on subsequent poor sleep quality, but not vice versa. Hence, we find that the longitudinal association between drinking and subsequent poor sleep is stronger than any potential association between sleep and subsequent drinking in middle to later adulthood.

Previous literature encompasses few similar longitudinal studies with which to compare our results. There are some population based studies that have investigated longitudinal associations between sleep and alcohol use from adolescence [36] to emerging adulthood [37] and later adulthood [12], but most studies in the field have focused on investigating individuals diagnosed with AUD. We were interested in shedding light on the relationship between sleep and alcohol drinking in the general population in adulthood, so as to better understand the underlying factors influencing the development of both insomnia and AUD.

Using data from twin pairs, we were able to investigate the role of shared familial factors underlying the associations between alcohol use and sleep. Importantly, within-pair analyses suggested that the cross-sectional associations between heavy drinking and poor sleep quality were not fully explained by genetic and environmental influences shared by the co-twins. These findings do not establish a causal effect of alcohol use on sleep quality, but they are a first step in this direction as they enable us to rule out the possibility of a spurious association due to familial background. These findings are novel, as we are not aware of earlier twin studies on the associations between alcohol use and sleep quality. However, earlier research has suggested shared genetic influences on a related sleep trait, namely diurnal preference and problematic alcohol use, including increased quantity and binge drinking [38].

Heavy alcohol consumption and binge drinking are obvious risk factors and potential symptoms of AUD. Our findings suggest that both of these drinking traits are cross-sectionally connected to poor sleep and longitudinally predictive of poor sleep. Even though engaging in risky drinking even over a long period of time does not necessarily equal AUD, it should be noted that the majority of AUD cases remain undiagnosed and thereby untreated [39]. Although we are unable to speculate on the reasons behind drinking in the current study, previous literature suggests that many cases of alcohol consumption can be explained by the self-medication framework [9, 40]. When alcohol is used to ‘treat’ personal problems, including sleep problems, symptoms of AUD might develop whilst being left unnoticed. It is well known that chronic misuse of alcohol contributes to severe health consequences. What is often ignored is how drinking contributes to chronic disease by increasing the risk of developing insomnia, other sleep disorders or comorbidities. Accordingly, the health outcomes associated with or mediated by alcohol drinking might be greater than expected by the general population.

All earlier evidence taken together points towards a multifactorial connection between sleep and alcohol including the different components of sleep. Whereas increased drinking appears to be predictive of poor sleep according to our findings, other components of sleep may be differently associated with other patterns and levels of alcohol use. Indeed, in the context of sleep, it is worth noting that different neurotransmitter systems control different aspects of sleep so that timing (chronotype), duration (homeostatic need for sleep) and quality of sleep have partially independent mechanisms [41]. These systems might underlie the reasons why we were unable to see a consistent pattern from increased drinking to short sleep duration similar to that from increased drinking to poor sleep quality.

Our study is among the very few longitudinal studies using a cross-lagged model to investigate associations between poor sleep and alcohol consumption. The model enables us to see how associations develop during the long follow-up period, whilst controlling for associations within different time points and stability across time. It is also worth highlighting the uniqueness of the Older Finnish Twin Cohort data. What makes it scientifically invaluable is not only the fact that it consists of twin participants but also its long-term follow-up period of 36 years and the comprehensiveness of the questionnaires. The Finnish Twin Cohort is representative of the population with e.g. mortality and cancer incidence being the same as in the general adult population [42, 43].

Our findings should be interpreted in the light of the following limitations. First, self-reported data always entails the risk of reporting bias. Second, the measurement of sleep quality (and sleep duration) in the current study is based on a single question that has been subjectively answered by the participants. This brings out the issue as to how accurately the answers to this question reflect the reality. Third, the way sleep quality was measured might partly explain why the cross-lagged analyses revealed a consistent pattern between drinking and sleep quality, but not between drinking and sleep duration. A single measure of sleep quality might therefore not track well with sleep duration. Another potential factor explaining this inconsistency is insufficient statistical power to detect these associations. Specifically, the within-pair analyses of MZ twins likely suffered from insufficient statistical power due to small samples, which limited the interpretation regarding genetic vs. shared environmental contributions. Fourth, despite the longitudinal setting, this study on its own cannot establish causality and should be interpreted in the light of earlier and accumulating literature. Finally, the oldness of the dataset may be regarded as a limitation, considering that societal norms and trends in alcohol consumption and sleeping habits have changed during the past decades.

## Conclusion

It has been well established that not getting enough good quality sleep increases the risk of disease. Our research implies that long-term alcohol drinking in adulthood predicts decreased sleep quality later in life, and that familial factors do not fully account for this association. We wish to highlight that consuming moderate and large amounts of alcohol might adversely influence the quality of sleep over time, thereby increasing the risk of developing chronic sleep problems and affecting overall health.

## Supplementary Material

zpac023_suppl_Supplementary_Material

## Data Availability

Data access is provided by the Institute for Molecular Medicine Finland (FIMM) Data Access Committee (DAC) (fimm-dac@helsinki.fi) for authorized researchers who have IRB/ethics approval and an institutionally approved study plan. To ensure the protection of privacy and compliance with national data protection legislation, a data use/transfer agreement is needed, the content and specific clauses of which will depend on the nature of the requested data. *Conflict of interest statement*. None declared.

## References

[1] Morin CM , et al Epidemiology of insomnia: prevalence, self-help treatments, consultations, and determinants of help-seeking behaviors. Sleep Med. 2006;7(2):123–130. doi:10.1016/j.sleep.2005.08.008. 16459140

[2] Kronholm E , et al Prevalence of insomnia-related symptoms continues to increase in the Finnish working-age population. J Sleep Res. 2016;25(4):454–457. doi:10.1111/jsr.12398. 26868677

[3] Lane JM , et al Biological and clinical insights from genetics of insomnia symptoms. Nat Genet. 2019;51(3):387–393. doi:10.1038/s41588-019-0361-7. 30804566 PMC6415688

[4] Sofi F , et al Insomnia and risk of cardiovascular disease: a meta-analysis. Eur J Prev Cardiol 2014;21(1):57–64. doi:10.1177/2047487312460020. 22942213

[5] Kok VC , et al Risk of autoimmune disease in adults with chronic insomnia requiring sleep-inducing pills: a population-based longitudinal study. J Gen Intern Med. 2016;31(9):1019–1026. doi:10.1007/s11606-016-3717-z. 27130621 PMC4978676

[6] Hertenstein E , et al Insomnia as a predictor of mental disorders: a systematic review and meta-analysis. Sleep Med Rev. 2019;43:96–105. doi:10.1016/j.smrv.2018.10.006. 30537570

[7] Colrain IM , et al Alcohol and the sleeping brain. Handb Clin Neurol 2014;125:415–431. doi:10.1016/B978-0-444-62619-6.00024-0. 25307588 PMC5821259

[8] Koob GF , et al Alcohol use disorder and sleep disturbances: a feed-forward allostatic framework. Neuropsychopharmacology. 2020;45(1):141–165. doi:10.1038/s41386-019-0446-0. 31234199 PMC6879503

[9] Roehrs T , et al Ethanol as a hypnotic in insomniacs: self administration and effects on sleep and mood. Neuropsychopharmacology. 1999;20(3):279–286. doi:10.1016/S0893-133X(98)00068-2. 10063488

[10] Ebrahim IO , et al Alcohol and sleep I: effects on normal sleep. Alcohol Clin Exp Res. 2013;37(4):539–549. doi:10.1111/acer.12006. 23347102

[11] Crum RM , et al Sleep disturbance and risk for alcohol-related problems. Am J Psychiatry. 2004;161(7):1197–1203. doi:10.1176/appi.ajp.161.7.1197. 15229051

[12] Britton A , et al The association between alcohol consumption and sleep disorders among older people in the general population. Sci Rep. 2020;10(1):5275. Published 2020 Mar 24. doi:10.1038/s41598-020-62227-0 32210292 PMC7093458

[13] Arnedt JT , et al Treatment options for sleep disturbances during alcohol recovery. J Addict Dis. 2007;26(4):41–54. doi:10.1300/J069v26n04_06. PMC2936493 18032231

[14] Brower KJ , et al Prevalence and correlates of withdrawal-related insomnia among adults with alcohol dependence: results from a national survey. Am J Addict. 2010;19(3):238–244. doi:10.1111/j.1521-0391.2010.00035.x. 20525030 PMC2998536

[15] Chakravorty S , et al Alcohol dependence and its relationship with insomnia and other sleep disorders. Alcohol Clin Exp Res. 2016;40(11):2271–2282. doi:10.1111/acer.13217. 27706838 PMC7486899

[16] Kaprio J , et al The older Finnish twin cohort - 45 years of follow-up. Twin Res Hum Genet. 2019;22(4):240–254. doi:10.1017/thg.2019.54. 31462340

[17] Hublin C , et al Changes in sleep quality with age-a 36-year follow-up study of Finnish working-aged adults. J Sleep Res. 2018;27(4):e12623. doi:10.1111/jsr.12623. 29047168

[18] Hublin C , et al Changes in self-reported sleep duration with age—a 36-year longitudinal study of Finnish adults. BMC Public Health. 2020;20(1):1373. doi:10.1186/s12889-020-09376-z 32907578 PMC7487757

[19] Virtanen S , et al Birth cohort effects on the quantity and heritability of alcohol consumption in adulthood: a Finnish longitudinal twin study. Addiction 2019;114(5):836–846. doi:10.1111/add.14533. 30569536

[20] U.S. Department of Agriculture and U.S. Department of Health and Human Services. Dietary Guidelines for Americans, 2020-2025. 9th Edition. December, 2020. https://www.dietaryguidelines.gov/sites/default/files/2020-12/Dietary_Guidelines_for_Americans_2020-2025.pdf. Accessed August 1, 2022.

[21] Dickerman BA , et al Alcohol intake, drinking patterns, and prostate cancer risk and mortality: a 30-year prospective cohort study of Finnish twins. Cancer Causes Control 2016;27(9):1049–1058. doi:10.1007/s10552-016-0778-6. 27351919 PMC5278639

[22] Koivumaa-Honkanen H , et al Life satisfaction and depression in a 15-year follow-up of healthy adults. Soc Psychiatry Psychiatr Epidemiol. 2004;39(12):994–999. doi:10.1007/s00127-004-0833-6. 15583908

[23] Guney S , et al Dimensions of mental health: life satisfaction, anxiety and depression: a preventive mental health study in Ankara University students population. Procedia - Social Behav Sci. 2010;2(2):1210–1213. doi:10.1016/j.sbspro.2010.03.174.

[24] Nes RB , et al Major depression and life satisfaction: a population-based twin study. J Affect Disord. 2013;144(1-2):51–58. doi:10.1016/j.jad.2012.05.060. 23021825 PMC3513516

[25] Koivumaa-Honkanen H , et al Self-reported life satisfaction and alcohol use: a 15-year follow-up of healthy adult twins. Alcohol Alcohol. 2012;47(2):160–168. doi:10.1093/alcalc/agr151. 22215005

[26] Williams RA . Note on robust variance estimation for cluster-correlated data. Biometrics 2000;56(2):645–646. doi:10.1111/j.0006-341x.2000.00645.x. 10877330

[27] Allison PD . Fixed effects regression models. Applications in the Social Sciences, No. 160. Los Angeles, Sage Publications; 2008.

[28] McGue M , et al Causal inference and observational research: the utility of twins. Perspect Psychol Sci 2010;5(5):546–556. doi:10.1177/1745691610383511. 21593989 PMC3094752

[29] Roehrs T , et al Sleep, sleepiness, sleep disorders and alcohol use and abuse. Sleep Med Rev. 2001;5(4):287–297. doi:10.1053/smrv.2001.0162. 12530993

[30] Stein MD , et al Disturbed sleep and its relationship to alcohol use. Subst Abus. 2005;26(1):1–13. doi:10.1300/j465v26n01_01. PMC2775419 16492658

[31] Stone BM . Sleep and low doses of alcohol. Electroencephalogr Clin Neurophysiol. 1980;48(6):706–709. doi:10.1016/0013-4694(80)90427-7. 6155259

[32] Miyata S , et al REM sleep is impaired by a small amount of alcohol in young women sensitive to alcohol. Intern Med. 2004;43(8):679–684. doi:10.2169/internalmedicine.43.679. 15468965

[33] Breslau N , et al Sleep disturbance and psychiatric disorders: a longitudinal epidemiological study of young adults. Biol Psychiatry. 1996;39(6):411–418. doi:10.1016/0006-3223(95)00188-3. 8679786

[34] Wong MM , et al Sleep problems in early childhood and early onset of alcohol and other drug use in adolescence. Alcohol Clin Exp Res. 2004;28(4):578–587. doi:10.1097/01.alc.0000121651.75952.39. 15100609

[35] Janson C , et al Insomnia in men-a 10-year prospective population based study. Sleep 2001;24(4):425–430. doi:10.1093/sleep/24.4.425. 11403527

[36] Pasch KE , et al Longitudinal bi-directional relationships between sleep and youth substance use. J Youth Adolesc 2012;41(9):1184–1196. doi:10.1007/s10964-012-9784-5. 22752878 PMC3431186

[37] Troxel WM , et al Longitudinal associations of sleep problems with alcohol and cannabis use from adolescence to emerging adulthood. Sleep 2021;44(10):zsab102. doi:10.1093/sleep/zsab102. 33884430 PMC8561242

[38] Watson NF , et al A twin study of genetic influences on diurnal preference and risk for alcohol use outcomes. J Clin Sleep Med. 2013;9(12):1333–9. doi:10.5664/jcsm.3282 24340296 PMC3836345

[39] Probst C , et al Alcohol use disorder severity and reported reasons not to seek treatment: a cross-sectional study in European primary care practices. Subst Abuse Treat Prev Policy. 2015;10:32. doi:10.1186/s13011-015-0028-z 26264215 PMC4534056

[40] Johnson EO , et al Epidemiology of alcohol and medication as aids to sleep in early adulthood. Sleep 1998;21(2):178–186. doi:10.1093/sleep/21.2.178. 9542801

[41] Rosenwasser AM . Functional neuroanatomy of sleep and circadian rhythms. Brain Res Rev. 2009;61(2):281–306. doi:10.1016/j.brainresrev.2009.08.001. 19695288

[42] Kaprio J . The Finnish Twin Cohort Study: an update. Twin Res Hum Genet. 2013;16(1):157–162. doi:10.1017/thg.2012.142. 23298696 PMC4493754

[43] Skytthe A , et al Cancer incidence and mortality in 260,000 nordic twins with 30,000 prospective cancers. Twin Res Hum Genet. 2019;22(2):99–107. doi:10.1017/thg.2019.10. 31020942

